# Sudden Sensorineural Hearing Loss Following the Second Dose of COVID-19 Vaccine

**DOI:** 10.7759/cureus.17435

**Published:** 2021-08-25

**Authors:** Nikolaos Tsetsos, Alexandros Poutoglidis, Konstantinos Vlachtsis, Adamantios Kilmpasanis, Spyridon Gougousis

**Affiliations:** 1 Department of Otorhinolaryngology - Head and Neck Surgery, “G. Papanikolaou” General Hospital, Thessaloniki, GRC

**Keywords:** covid-19, sudden sensorineural hearing loss (ssnhl), covid-19 vaccine, sensorineural deafness, acetylosalicid acid

## Abstract

Sudden sensorineural hearing loss (SSHL) is a common otolaryngology emergency that significantly affects the patient’s quality of life. Although in most cases its etiology remains unknown (idiopathic SSHL), viral infections and vascular compromise constitute the most widely accepted etiopathogenic mechanisms. Specifically, occlusion of the internal auditory artery has been reported in cases of sudden deafness. Thrombotic events following the Oxford-AstraZeneca COVID-19 vaccine are rare. There have been reports of SSHL following immunization with Pfizer and Moderna COVID-19 vaccine; however, no etiologic relationship has been established between the two entities yet. We present a unique case of SSHL following the second dose of the Oxford-AstraZeneca COVID-19 vaccine. A 61-year-old female was referred to our department with a four-day history of the right-sided sense of fulness combined with almost complete hearing loss that had started two days after the second dose of Oxford-AstraZeneca COVID-19 vaccine. Pure tone audiometry showed profound right-sided sensorineural hearing loss. Magnetic resonance imaging of the brain and internal auditory canal and magnetic resonance angiography were both normal. The combination of glucocorticoids and acetylsalicylic acid leads to almost full recovery 15 days after deafness. The COVID-19 era is full of new challenges and clinical dilemmas. In our case, the addition of acetylosalicid acid to the patient’s initial treatment may have contributed to the hearing restoration; however, this fact will remain a hypothesis.

## Introduction

Sudden sensorineural hearing loss (SSHL) is defined as the rapid onset of hearing of at least 30 dB across three consecutive frequencies occurring within a period of 72 hours based on the US National Institute for Deafness and Communication Disorders (NICDC) [1]. Its annual incidence ranges from 5 to 20 cases in 100,000 individuals, with an estimated number of 66,000 new cases per year in the USA [2]. Viral infections, vascular compromise, and autoimmune disorders are some of the possible etiologies, although in 90% of the patients, no cause is identified despite the exhaustive investigation, and the SSHL is characterized as idiopathic [2].

Vascular compromise due to hemorrhage, thrombosis, and vasospasm in the inner ear vessels may lead to cochlear ischemia and potentially SSHL [3]. However, current guidelines advise against the use of thrombolytics, vasodilators, or vasoactive agents as routine therapy in patients with SSHL, due to the lack of documented efficacy and the potential side effects [2].

## Case presentation

A 61-year-old female was referred to our Department with a four-day history of the right-sided sense of fulness combined with almost complete hearing loss that had started two days after the second dose of Oxford-AstraZeneca COVID-19 vaccine. She did not report any history of earache, trauma, or discharge. Hearing loss was not accompanied by tinnitus or vertigo. She had a medical history of hypertension, dyslipidemia, and Hashimoto's thyroiditis all under medical treatment. None of her prescribed medication had been commenced within the previous six months. She denied tobacco use or alcohol consumption.

**Figure 1:**
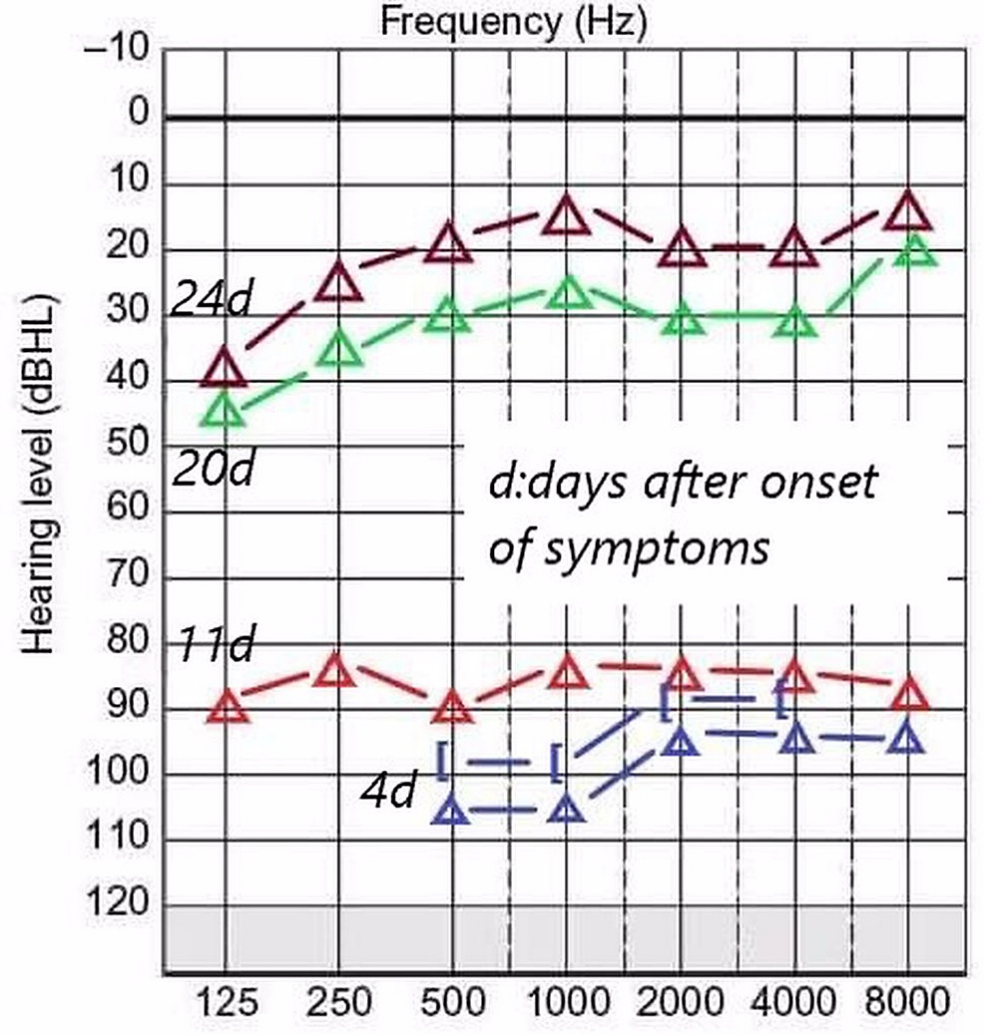
Pure tone audiogram presenting the course of hearing improvement from day 4 after the onset of symptoms (admission in the ENT Department) to day 24 (completion of oral glucocorticoids and acetylsalicylic acid).

Otoscopy was unremarkable on both sides. Weber tuning fork test lateralized to the left ear while Rinne test was positive on both sides. Pure tone audiometry showed profound right-sided SSHL of at least 85 dB in every frequency (250-8,000 Hz) (Figure 1).

The initial routine diagnostic workup did not reveal any abnormal findings.

**Figure 2:**
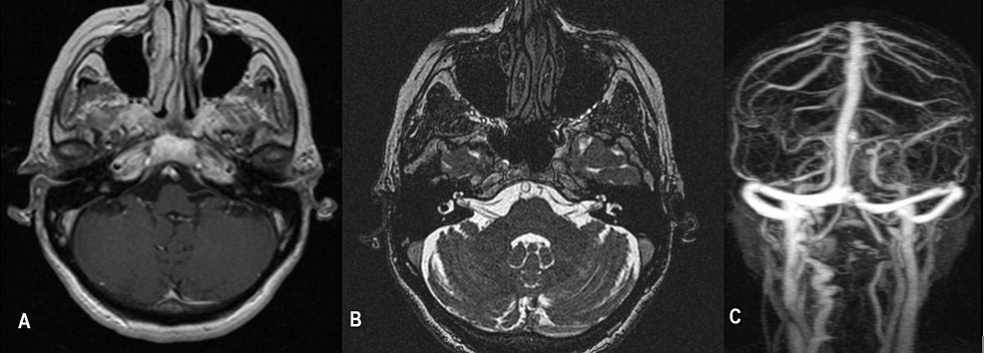
(A) Postcontrast axial T1WI MRI (B) Postcontrast axial T2WI MRI and (C) MRA scan of the circle of Willis excluded any identifiable cause of the unilateral hearing loss.

The patient received systemic intravenous glucocorticoids (Dexamethasone 8 mg three times per day for three days with tapering over a week). However, due to her recent history of vaccination with Oxford-AstraZeneca COVID-19 vaccine, neurologists and internists were asked to assess the patient. Anticoagulant therapy with 100 mg of acetylsalicylic acid once daily was commenced. The hearing was evaluated every two days with new pure tone audiometry, which did not reveal any remarkable improvement. One week after treatment initiation, magnetic resonance imaging (MRI) of the brain and internal auditory canal and magnetic resonance angiography (MRA) were conducted. Both imaging tests were normal (Figures 2A-2C).

The patient was discharged from the hospital after seven days and was prescribed a nine-day course of oral glucocorticoids and acetylsalicylic acid. After completing the treatment, the patient returned to our Audiology Clinic for a new pure tone audiogram. The patient reported significant improvement in her hearing (15 days after deafness onset and 11 days after initiation of treatment). The new audiogram showed a significant increase in her threshold (almost full recovery) (Figure 1).

## Discussion

The cochlea is mainly supplied by one terminal artery, the labyrinthine artery, something that renders it very sensitive to circulatory alterations and ischemia. Thrombosis or vasospasm of the internal auditory artery constitutes one of the main etiopathogenetic mechanisms for idiopathic SSHL. However, the location of the inner ear and its blood supply in the temporal bone, allowed only experimental studies to advocate this mechanism [4].

AstraZeneca COVID-19 vaccine (ChAdOx1 nCoV-19, AZD1222, Vaxzevria; Oxford/AstraZeneca) provides effective immunization against SARS-CoV-2 in the general population [5]. However, safety concerns about increased risk for thrombotic events have led to a temporary halt of vaccination with this vaccine by the German Ministry of Health and other European countries [6,7]. The percentage of deaths following thrombosis is very low and it is suggested that the benefits of immunization far outweigh the risks of potential side effects [8].

In the USA, a Johns Hopkins study concluded that COVID-19 vaccines are not associated with an increased risk for SSHL, based on the evaluation of 40 cases (28 had received the Pfizer vaccine, and 12 had received the Moderna vaccine) of confirmed SSHL among 86,553,330 vaccinations. The incidence of SSHL following the COVID-19 vaccination was estimated to be 0.3% and not only did it not exceed that of the general population, but it could be deemed lower [9]. However, this study is limited by the fact that no other vaccines that are now widely used such as the Oxford-AstraZeneca covid-19 vaccine, were included. Our plan to add acetylosalicid acid to the patient’s initial treatment may have contributed to the hearing restoration; however, this fact will remain a hypothesis.

## Conclusions

The COVID-19 era is full of new challenges and clinical dilemmas. A case of SSHL two days after the COVID-19 vaccination should be evaluated carefully and should not be dismissed as a coincidental event. The addition of acetylosalicid acid to the patient’s initial treatment may have contributed to the hearing restoration; however, this fact will remain a hypothesis, as no objective test can ascertain the occlusion of such a microcirculation.
